# A novel way of determining gestational age upon the birth of a child

**DOI:** 10.7189/jogh.11.03078

**Published:** 2021-09-04

**Authors:** Kumanan Wilson, Victoria Ward, Pranesh Chakraborty, Gary L Darmstadt

**Affiliations:** 1 Department of Medicine, University of Ottawa, Ottawa, Ontario, Canada; 2 Clinical Epidemiology Program, Ottawa Hospital Research Institute, Ottawa, Ontario, Canada; 3 Bruyère and Hospital Research Institutes, Ottawa Ontario, Canada; 4 Department of Pediatrics, Stanford University School of Medicine, Stanford, California, USA; 5 Department of Pediatrics, Children’s Hospital of Eastern Ontario and University of Ottawa, Ottawa, Ontario, Canada; 6 Newborn Screening Ontario, Ottawa, Ontario, Canada

Preterm birth is the leading cause of infant morbidity and mortality globally [1]. Determining whether an infant was born preterm can be challenging, especially in low-resource settings due to a paucity of prenatal care and dating ultrasounds, the unreliability of recall of last menstrual period, and recognised limitations of Ballard score and other newborn clinical assessment and anthropometric measurements [2]. Accurate estimation of gestational age (GA) is important in informing the medical care of the newborn and accurately assessing neurocognitive development. GA dating is also necessary for population-level determination of preterm birth rates as well as appropriate resource and intervention allocations. There is a need for better data that provide robust estimates of the burden of preterm birth in low-resource settings.

## OPPORTUNITIES FROM METABOLIC GA TESTING

Recently, novel methods for establishing an infant’s GA have emerged that may be able to overcome some of the existing limitations. One such approach involves using an established public health procedure, a heel prick blood spot typically used for newborn screening [3]. Newborn screening is a routine practice in many high-income countries, wherein a blood spot is collected via heel prick from newborns to screen them for a variety of rare, treatable conditions. The blood spot is analysed to determine levels of several analytes including acylcarnitines, amino acids, and endocrine markers. Abnormalities in specific analytes or ratios of analytes may suggest an underlying disorder. Infants who are identified as “screen positive” for one or more of the screened conditions undergo confirmatory testing and, ideally, receive prompt, definitive treatment.

Levels of the analytes measured by newborn screening vary by the GA of an infant at birth [4]. While this can impact the performance of newborn screening, it has also created an opportunity to use the levels of these analytes at birth to determine the infant’s GA. Three separate groups in Canada and the United States have successfully created algorithms that use birthweight and analytes from their newborn screening programmes to accurately estimate GA within one to two weeks [4-6]. The addition of fetal-to-adult hemoglobin ratio, measured when screening for hemoglobinopathies, has further improved the predictive ability of these algorithms [7]. Preliminary data from studies seeking to validate these algorithms in low-resource settings have shown promising results and suggest potential utility for these models on cord blood samples in addition to samples derived from heel prick.

**Figure 1:**
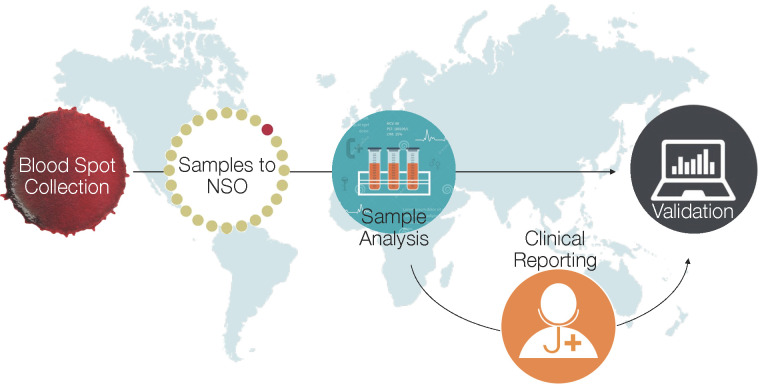
Gestational age assessment in international preterm birth cohorts.

Further validation of this approach is necessary to determine to what extent local factors may affect metabolic profiles, which in turn could impact the accuracy and generalisability of metabolic-derived estimates of GA. We are currently in the process of implementing and further evaluating this approach to GA estimation in multiple low-resource settings in sub-Saharan Africa and South Asia, including Kenya, Zambia, Zimbabwe, and Bangladesh [8]. Heel prick and/or cord blood samples are being obtained from infants in these sites and sent to Newborn Screening Ontario laboratories for further analysis. This approach will generate population-level estimates of rates of preterm birth and small-for-gestational age (SGA) infants, ultimately intended to inform global and regional estimates of the burden of preterm birth. Our approach also includes screening for select metabolic disorders which can be successfully followed up and treated at the sites [9] (**Figure 1**). The University of California – San Francisco has completed a similar study in Uganda [10].

## CHALLENGES TO THIS APPROACH

Logistical challenges with obtaining samples within an appropriate timeframe (ideally 24-72 hours after birth) and then shipping them to an external laboratory under conditions that preserve the quality of the blood spots are important to address. Ultimately, the goal will be to develop in-country capacity and point-of-care diagnostics that will permit these analyses at the time of birth to inform care for individual infants. There are also cultural concerns to be understood and addressed that may affect willingness of families to provide either heel prick or cord blood samples. Another challenge is the adaptation of algorithms developed using blood spot data to estimate population rates of SGA. Existing algorithms can be enhanced using machine learning approaches to improve model performance, and could be further reiterated for SGA-specific estimations [11].

Is this approach worth the cost? According to our estimates, the incremental cost per preterm infant correctly classified by the metabolic algorithm is US$11 542, and per SGA infant is US$688 [12]. Determining whether these costs are acceptable in resource poor settings is an important consideration. While birth weight alone is reasonable at identifying appropriate-for-gestational age (AGA) preterm infants, our analyses suggest that the real value of this approach may be to help identify SGA infants and distinguish them from preterm infants. Ultimately, the value of this approach is dependent on the reduction in morbidity, mortality and health resource utilisation that this identification can realise.

To optimise benefit for costs, additional potential value to the collection of these samples should be evaluated. An immediate obvious benefit is the potential to guide the care of appropriately recognised preterm infants, provided that metabolic data can be analysed and processed promptly. Moreover, the analysis of the samples can provide newborn screening value by identifying infants at risk of treatable conditions such as congenital hypothyroidism and hemoglobinopathies.

Looking to the future, work is under way to determine whether there is additional value to the analysis of blood spots, including their predictive value for a host of conditions including early neonatal mortality and neonatal sepsis [13,14]. Blood spots can also potentially be used for genetic analyses. Expanded metabolomic panels that go beyond the analytes collected for newborn screening purposes may also improve the predictive value of the blood spots for identifying modifiable risk factors and treatable conditions. In addition, the predictive and diagnostic utility of newborn blood spots may be expanded through integration of data derived from maternal blood and maternal health history [15].

**Figure:**
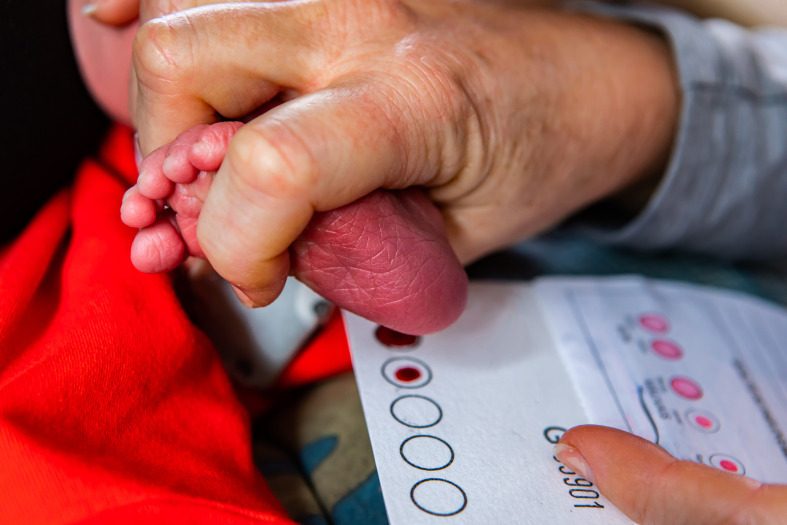
Photo: Close up details as a family doctor carries out a heel prick blood test during newborn baby health screening, squeezing drops from foot for analysis (Valmedia, via: https://www.shutterstock.com/image-photo/close-details-family-doctor-carries-out-1628211025).

## CONCLUSION

We envision a future where a heel prick at birth could inform caregivers of the GA of a child, distinguish SGA from preterm infants, identify treatable conditions and determine risk for specific health conditions. This data could also provide population-level estimates of preterm birth to guide health system planning and resource allocation for improved cost-effectiveness and impact of maternal and child health policies and programmes.
